# Malaria elimination in Isabel Province, Solomon Islands: establishing a surveillance-response system to prevent introduction and reintroduction of malaria

**DOI:** 10.1186/1475-2875-10-235

**Published:** 2011-08-11

**Authors:** Matthew O'Sullivan, Geoffrey Kenilorea, Yuka Yamaguchi, Albino Bobogare, Landry Losi, Jo-An Atkinson, Andrew Vallely, Maxine Whittaker, Marcel Tanner, Rushika Wijesinghe

**Affiliations:** 1 Pacific Malaria Initiative Support Centre, Australian Centre for International and Tropical Health, School of Population Health, University of Queensland, Brisbane, Australia; 2 National Vector Borne Disease Control Programme, Ministry of Health, Honiara, Solomon Islands; 3 National Centre in HIV Epidemiology and Clinical Research, University of New South Wales, Sydney, Australia; 4 Swiss Tropical and Public Health Institute, Basel, Switzerland

## Abstract

**Background:**

The Solomon Islands National Malaria Programme is currently focused on intensified control and progressive elimination. Recent control efforts in Isabel Province have reduced their malaria incidence to 2.6/1,000 population in 2009 [1] whereas most neighbouring provinces have much higher incidences. A malaria surveillance-response system that involves testing all travellers entering Isabel Province using rapid diagnostic tests (RDT) to prevent cases being imported had been proposed by local health authorities. This study provides information on the feasibility and acceptability of implementing a new approach of surveillance and response in the context of low levels of indigenous malaria transmission in Isabel Province.

**Methods:**

A total of 13 focus group discussions (FGD) and 22 key informant interviews (KII) were conducted in Isabel Province, Solomon Islands. Key topics included: the travel patterns of people to, from and within Isabel Province; the acceptability, community perceptions, attitudes and suggestions towards the proposed surveillance programme; and management of suspected malaria cases. This information was triangulated with data obtained from port authorities, airlines and passenger ships travelling to and from Isabel Province in the preceding two years.

**Results:**

Travel within Isabel Province and to and from other provinces is common with marked seasonality. The majority of inter-provincial travel is done on scheduled public transport; namely passenger ships and aircrafts. In Isabel Province there is a healthy community spirit as well as high concern regarding malaria and its importation and there is currently effective malaria passive case detection and management. Conducting malaria screening at ports and airports would be acceptable to the community.

**Conclusion:**

A robust surveillance-response system is essential when moving towards malaria elimination. Many factors contribute positively towards the feasibility of an RDT based malaria surveillance system in Isabel Province. Due to financial and logistical restraints local health authorities have concluded that a system of community-based vigilance to identify new arrivals in villages and direct them to have malaria testing is more feasible than formal screening at ports and airports. A surveillance response system to prevent introduction of malaria into Isabel Province can be integrated into the National Malaria Control Programme provided the operational steps are carefully planned with regards to human and financial resources.

## Background

**Figure 1:**
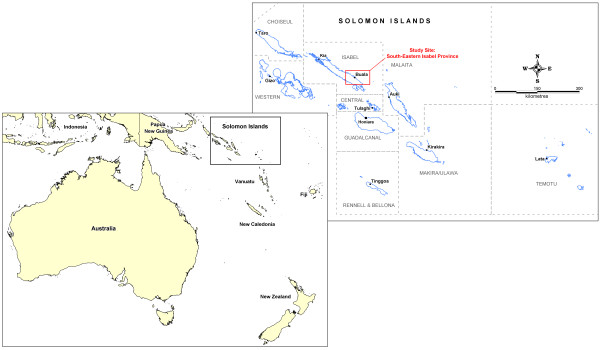
**Map of the Solomon Islands**.

Solomon Islands (SI) (Figure 1) ranks highly amongst the countries most affected by malaria in the Asia-Pacific region, and in terms of morbidity and mortality malaria is a major public health problem [2,3]. However, there have been recent improvements with the national Annual Parasite Incidence (API) Rate decreasing from 131/1,000 in 2007 to 77/1,000 in 2009 [1,4]. In March 2008, Solomon Islands government upgraded the goal of the National Malaria Control Programme to intensified control and progressive elimination [5,6]. Monitoring, evaluation and surveillance have become an important and integral part of the programme that require special attention as malaria prevalence is decreasing and elimination becomes a reality for certain provinces.

**Figure 2:**
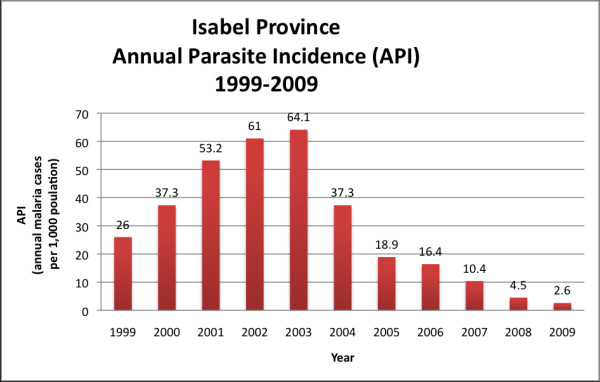
**Annual Parasite Incidence Rate in Isabel Province from 1999 to 2009 **[7]. (Data from the SI Malaria Information System (MIS) based on laboratory confirmed malaria)

Malaria control efforts in Isabel Province have been particularly successful in recent years. The Annual Parasite Incidence (API) Rate in Isabel Province has steadily declined from 64.1 per 1,000 population in 2003 to 2.6 per 1,000 population in 2009 (Figure 2) [7].

**Figure 3:**
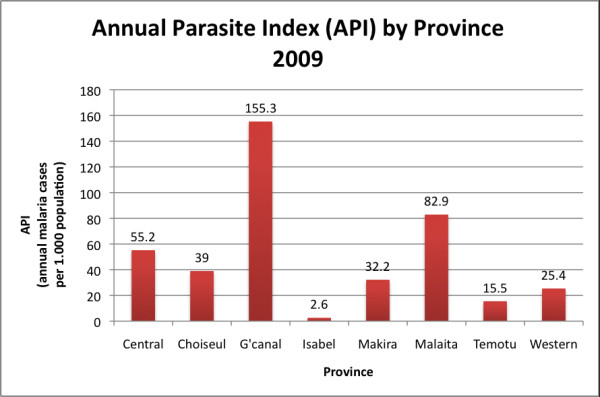
**Annual Parasite Incidence Rate per 1,000 population by Province in 2009 **[1]. (Data from the SI Malaria Information System (MIS) based on laboratory confirmed malaria)

The observed reductions in incidence were corroborated by a mass blood survey in October 2009 that found only one positive slide (*Plasmodium falciparum*) out of 8,600 tested by microscopy and four positive samples (three *P. falciparum *and one *Plasmodium vivax*) out of 2,071 tested by polymerase chain reaction (PCR) [8]. Surrounding provinces have been less successful in their control efforts and their malaria incidence remains relatively high (Figure 3). The SI government has officially endorsed the targeting of Isabel and Temotu Provinces for malaria elimination as a result of their successful respective control programmes which have reduced local transmission to low levels [9].

It is well established that epidemics occur as a result of migration of people from malaria endemic areas to areas with low or no transmission but with efficient vectors [10,11]. It is argued that the failure to consider population mobility in the ambitious Global Malaria Eradication Programme of the 1950s and 1960s contributed to the inability to maintain the gains that were made in a number of developing countries [12,13]. Therefore, with renewed global interest for malaria elimination, the importance of a robust surveillance-response system at points of entry from areas of higher transmission, that entails swift treatment and follow-up of infected individuals and their environment, has been recognized [14].

With low malaria incidence having been achieved, Isabel Province has moved to a phase of elimination known as 'holding the line' (i.e. to maintain or further reduce this level and prevent the introduction of malaria cases from surrounding areas) [14]. A surveillance-response system to prevent malaria cases being imported into Isabel Province from nearby high transmission areas by testing all travellers using malaria Rapid Diagnostic Test (RDT) kits has been proposed by local health authorities. Internal surveillance as part of scheduled transport between provinces geographically suits island nations as opposed to mainland countries [14]. However, there is minimal information available regarding the feasibility and acceptability of implementing such a programme in the region. Thorough investigation and assessment of the situation on the ground is essential for such a surveillance-response system to be successful. An assessment of the magnitude, methods and patterns of population movement is required [12]. Epidemiological research in the Papua New Guinea highlands has shown that that the mobility and remoteness of semi-nomadic populations in south Simbu has greatly reduced the effectiveness of indoor residual spraying (IRS) [15]. No study to date has thoroughly examined population mobility in association with malaria transmission in Solomon Islands. As well as collecting practical, operative and quantitative information, it is also important to ascertain community perceptions of such surveillance measures and through collaboration and partnership building, design a system that is both acceptable to communities and sustainable by local health authorities [16].

This study was carried out at the request of the National Vector Borne Disease Control programme (NVBDCP), SI to provide information to the country health managers regarding the feasibility and acceptability of various malaria surveillance interventions to prevent introduction and reintroduction of malaria to Isabel Province. It entailed ascertaining facilitators and barriers likely to influence future programme success, including the movement patterns of people into and out of Isabel Province as well as community-level perceptions and acceptability of an RDT-based surveillance-response system.

## Methods

### Study location and population

This study was conducted in Isabel Province (Figure 1) from March to April 2010. Isabel Province is approximately 80 km north-west of Guadalcanal Province and consists of Santa Isabel island and multiple smaller surrounding islands. In 2008 the Province had an estimated population of 26,221 [7] of the country's total population of 510,600 [17].

Most of the island is rugged terrain, with lush rainforest and a mountain range running down its centre [18]. The climate is tropical and has an average daytime temperature of 29°C which varies little throughout the year [18].

Buala village is the capital of Isabel Province and is located on the coast at the eastern end of the Island. The majority of the provincial population resides in the south-east of the island; however Kia in the north of the island is the largest village and Buala is the second largest [18]. Overland transport infrastructure is minimal and the interior can only be reached via foot tracks. Most villages lie on the coast and villagers usually rely on boats for transport. Primary occupations include agriculture, fishing, hunting, and domestic duties. Forestry and logging are also significant industries on the island. Many of those working in subsistence activities are unpaid [18].

Isabel Province has developed a unique contemporary local leadership structure known as 'the Tripod', a longstanding cooperative alliance between Village Chiefs, the Church and the Provincial Government [19] which was established due to the strong social influence of the Chiefs and the Church in the community [18]. The Mothers' Union, a worldwide Anglican Church charitable society, plays an important social and spiritual role for the people in Isabel Province [20].

The 49 bed Provincial Hospital is located in Buala and is the only hospital on the island. The services in the hospital include a pharmacy, a small pathology laboratory, an X-ray machine, a health education unit, an environmental health unit and the malaria control team. The rest of the province is serviced by village health centres, rural clinics and nurse aid posts. Due to a lack of health and road infrastructure some remote villages have poor access to health services [18].

A convenience method was used to select villages located in the south-east region of the island (Figure 1). With counsel from a team of in-country public health and research officers, five 'typical' villages were selected. For purposes of the study these villages were labelled A, B, C, D and E to maintain participant anonymity.

### Study procedure

To enable triangulation of data a variety of qualitative research tools were used, including Focus Group Discussions (FGDs), Key Informant Interviews (KIIs) and informal field observation. In addition, airline and shipping records of flights and ships that operate between Isabel Province and Honiara were obtained from the relevant companies in Honiara. Data collection was carried out by one female and two male facilitators (YY, MO, GK). Facilitators underwent a three-day intensive training workshop in Honiara on qualitative research methods, ethics, data management and project logistics. This workshop was carried out in-country by the School of Population Health (SPH), University of Queensland (UQ) and was linked to the training of staff from the Solomon Islands Development Trust (SIDT) for capacity building purposes. Facilitators were supported in the field jointly by a provincial malaria officer and experienced staff from SPH, UQ.

Community leaders informed the village members of the FGD and KII and facilitated the recruitment of participants to the study. KIIs were carried out with influential community members including village chiefs, teachers, women's group activists, religious leaders, Provincial Council members and health care workers.

Separate FGDs were carried out for men and women where possible to allow diversity of opinions and to maximize engagement by all participants [21]. The FGDs and KIIs were conducted by the trained facilitators and supported by an interpreter as required. All interviews were conducted in SI Pijin or in English and recorded using a digital recorder, backed up with manual note taking. Structured interview guides were used to direct discussions, however participants were encouraged to discuss their own views and opinions with minimal interruption and each discussion lasted between one to two hours.

Three main topics were discussed; travel habits (where to, how frequently and reasons for travel), malaria surveillance (risk of importation of malaria into the island, ideas for prevention of malaria importation and screening, and the acceptability of a proposed surveillance system) and the management of fever and suspected malaria cases in the community and in healthcare facilities (to ascertain management of potential cases undetected by surveillance).

During the KII and FGDs, a map of Isabel and nearby provinces was used to encourage interaction, discussion and to help gain a better understanding of the participants' travel habits. Participants were asked to draw the common routes of travel on a map and photographs of the maps were later used for interpretation and comparison. At the end of the FGDs and KIIs each participant's age, education, occupation, number of children (if relevant) and religion were recorded.

Data of passenger travel between Isabel Province and Honiara from the preceding two years was obtained from the Isabel Development Company (IDC), the provincial passenger shipping company, and Solomon Airlines. The data obtained was analysed to ascertain any distinct patterns of human movement at different times of the year.

### Analysis

All digital recordings were transcribed (and translated from Pidgin to English if necessary) by the field research team. The researchers received training on the use of Nvivo 8™ software (QSR International Pty Ltd, Australia) and on the analysis of qualitative data at UQ by SPH staff. An agreed coding key was developed by the research team using the interview guides, sample transcript reviews and discussion of coding issues which was then utilized by the primary coder. The data was organized into identifiable themes and was further analysed using Nvivo 8™. Analysis included the examination of differences in responses between villages and gender.

### Ethical considerations

Ethical approval for this study was obtained by the School of Population Health Ethics Committee, UQ, Australia, and the National Health Research Ethics Committee, Solomon Islands. Written informed consent was obtained from the chiefs of each selected village. All participants were required to be over 18 years of age. Verbal and written information was provided to participants regarding the study aims and procedures and individual consent (written or witnessed thumb print) obtained from all participants. Additional informed consent was obtained for the digital recording of interviews.

Demographic data received from participants was used only to assist the analysis and at an aggregated level. Wherever possible potential identifying data was de-identified prior to storage and participants were registered by study number rather, than personal identifiers to maintain confidentiality.

## Results

A total of 13 FGDs and 22 KIIs were carried out. FGD size varied from six to 12 participants. FGDs were conducted with men and women separately except for one FGD with healthcare workers and one with members of the Isabel Provincial Assembly both of which contained men and women to have sufficient numbers and this did not appear to inhibit open discussion.

The demographic profile of participants was relatively homogeneous except that Key Informants on average achieved a higher level of education (Table 1). Most participants were married and were affiliated with the Church of Melanesia.

**Table 1:** Demographic profile of study participants

		Key Informants	Focus Group Participants
		
		22 (17%)	108 (83%)
**Age Group**	**18-29**	2 (9%)	25 (23%)
	
	**30-49**	10 (45%)	51 (47%)
	
	**> 50**	8 (36%)	26 (24%)
	
	**Not indicated**	2 (9%)	6 (6%)

**Gender**	**Male**	18 (82%)	57 (53%)
	
	**Female**	4 (18%)	51 (47%)

**Religion**	**Church of Melanesia**	19 (86%)	92 (85%)
	
	**Other Christian**	2 (9%)	1 (1%)
	
	**Not indicated**	1 (5%)	15 (14%)

**Marital Status**	**Married**	20 (91%)	96 (89%)
	
	**Unmarried**	1 (5%)	10 (9%)
	
	**Living together**	0 (0%)	1 (1%)
	
	**Not indicated**	1 (5%)	1 (1%)

**Education**	**None**	0 (0%)	2 (2%)
	
	**Primary**	2 (9%)	27 (25%)
	
	**Secondary**	9 (41%)	45 (42%)
	
	**Tertiary**	9 (41%)	24 (22%)
	
	**Not indicated**	2 (9%)	10 (9%)

The study villages were generally homogeneous with regards to patterns of population mobility, community attitudes, perceptions, acceptability towards and suggestions regarding malaria surveillance. Participant responses differing between study villages, gender or participant groups are highlighted in the results presented, otherwise similar responses have been reported collectively. The IDC was only able to provide data of the number of passengers travelling by ship during January to June 2008 and for the month of November 2009. Reliable flight data of the number of passengers travelling by air was available from January 2008 to December 2009 from Solomon Airlines.

### Travel patterns

Buala is the most commonly visited destination within Isabel Province. Participants reported travelling by foot, by paddle canoe or by out-board motor boat (OBM). Those from villages further away often reported using the passenger ship more often.

**Figure 4:**
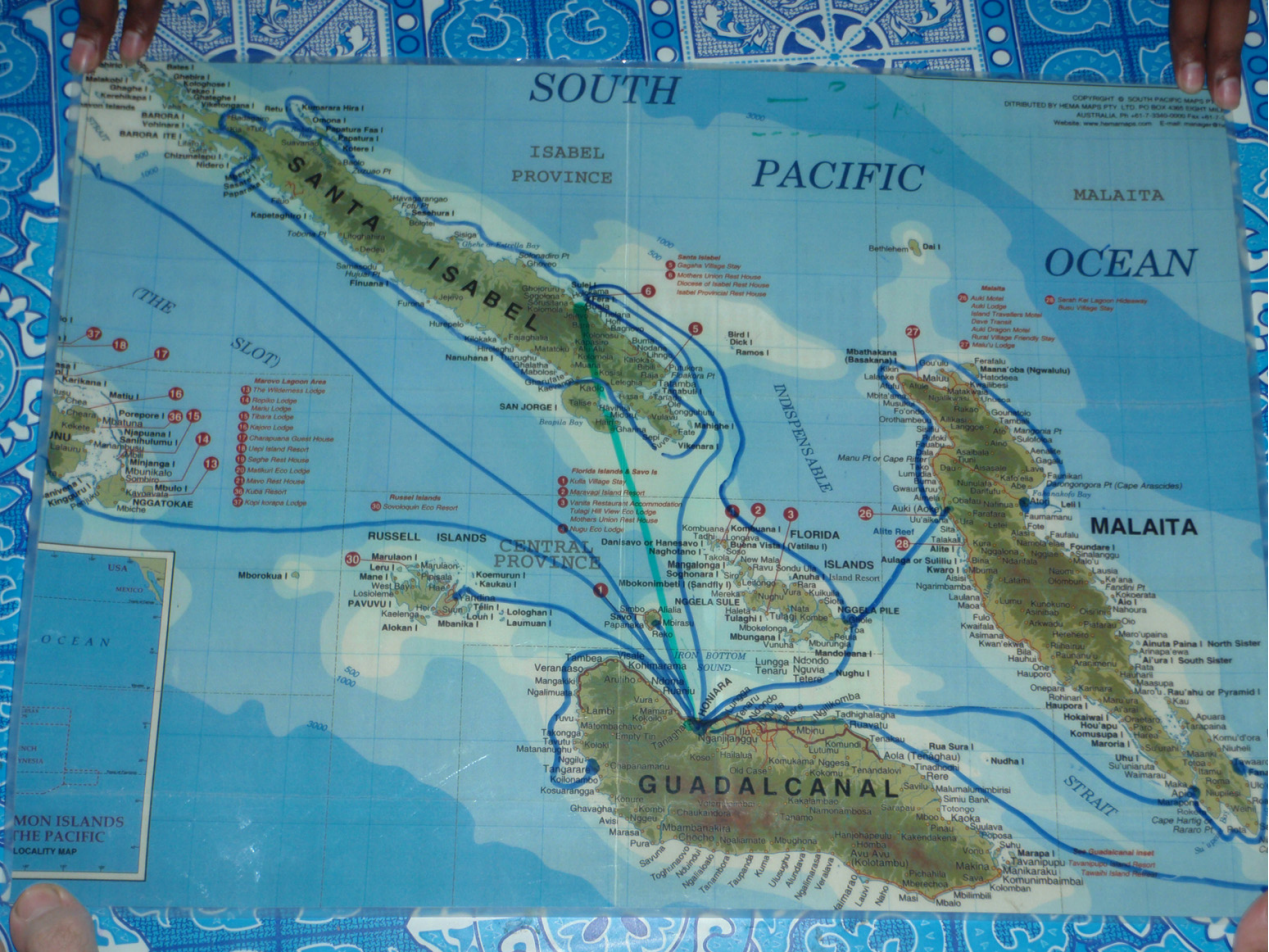
**Recent travel as indicated by male participants from a village in Isabel Province (blue - boat travel, green - air travel)**.

The most commonly reported destination visited outside of Isabel Province was Honiara, in Guadalcanal. Honiara is the central transport hub through which all scheduled public transport travels from Isabel Province before travelling to other provinces. Many participants reported travel to other provinces (Figure 4). Passenger ferry and scheduled flights are the principal modes of transport to and from other provinces and are the only forms of scheduled public transport available.

### Scheduled public transport

Passenger ships operated by the IDC travel from Honiara to each of three different island 'zones' and back once a week, stopping at 13 to 20 villages on each trip depending on the zone visited. Passenger ship was reported by all participants for travel to distant villages in Isabel Province including villages on the other side of the island and also for travel to Honiara and onwards to other provinces. Many participants reported the affordability of this mode of transport as a major determinant of its use.

*'Travelling by ship is cheaper than using out board motor engines which is very costly in terms of petrol' *(Male key informant, village A)

There are two airports in Isabel, namely Fera (servicing the south) and Suavanao (servicing the north). A 19 seat Twin Otter aircraft makes four flights a week from Honiara to Isabel Province (as at April 2010). FGD participants from the villages rarely reported using air travel; however key informants reported using air travel frequently to go to Honiara or beyond.

*'I would say business people (travel by aeroplane), but then again there's hardly anyone like that in our village. The only person I know who sometimes travels by air is the chief.' *(Male participant, FGD, Village E)

Most participants reported that travel by passenger ship and by air during holiday periods (Christmas, June and Easter) increases greatly. Records from the shipping company, IDC and airline data corroborated this.

### Unscheduled private transport

Due to the coastal location of most villages and limited road network, OBM are the main mode of transport and are usually only used over short distances. However it was reported that OBM is frequently used for longer travel by people in other regions of the province, such as Kia village in the north, to travel to Choiseul and Western Provinces and by people in the south to travel to Central and Guadalcanal Provinces. One participant identified this type of travel as a potential malaria importation risk:

*'(They sell) fish at markets in Honiara, spend two or three nights..........and then come again, every week, every week, see so it's likely that the malaria can come here'*. (Male participant, FGD, village A)

It was also often reported that OBM is frequently used by people from Kia village and elsewhere in the northern region to travel to Choiseul and Western Provinces and by people from the southeast region of Isabel Province to travel to Guadalcanal and Central Province to visit relatives. Most participants reported that there is increased travel by OBM during holiday periods. However, the availability of OBMs and the price of petrol were the main barriers to their use reported by many participants.

### Infrequently used modes of transport

Very few participants reported motor vehicle use and there were only two to three vehicles in Buala at the time of the study. There are a small number of people who travel infrequently between provinces by cargo ship or by helicopter. There is a small amount of tourism in Isabel Province and tourists usually travel on scheduled flights.

### Logging camp workers and transport

Most participants as well as the Department of Forestry reported extensive logging operations in Isabel Province. Logging camp workers usually travel by passenger ship and then transfer to the camps by logging company OBM. They are also sometimes transported directly to the camps by the logging company barge directly from other provinces which visit logging camps once or twice a month.

### Community attitudes and suggestions regarding malaria surveillance

Only a few participants were familiar with RDT therefore facilitators explained the use of RDT for malaria screening to all. A surveillance system to prevent malaria cases being imported into Isabel Province by testing all travellers using RDT had been proposed by local health authorities. Many participants proposed the idea of screening travellers entering their province for malaria before the topic was even introduced by facilitators.

### RDT testing on scheduled public transport

Participants were asked about their opinions towards routine RDTs being performed on all passengers on flights and passenger ships. Almost all participants accepted suggestions of routine RDTs before entering Isabel Province and said that they would be compliant towards testing. Participants were asked for suggestions about how, when and where testing should take place (Table 2). Only one key informant indicated that he would not be happy to have testing carried out on the ship due to stigma.

*'........if I heard that I had malaria, all the people on the ship will discriminate (against) me, or they will talk about me..... I would feel neglected or feel shame.......I think I have to refuse (the test).' *(Male key informant, village D)

**Table 2:** Participant suggestions for surveillance in Isabel Province to prevent importation of malaria

Suggestion:	Suggested by:	Advantages and disadvantages raised by participants:
RDT at wharf in Honiara before boarding ship	Most	Could potentially link with ticket purchase Could potentially be made a mandatory requirement before boarding

RDT onboard ship en route	Some	Prevents further boarding delay at already chaotic wharf May be less likely to miss people Overcrowding, minimal shelter and poor weather would hamper testing Potential stigma

Testing a day or two prior to departure	A few	Prevents further boarding delay at already chaotic wharf Passenger can contract malaria between testing and departure

Identifying mark to identify those tested or those testing positive (eg. indelible ink)	A few	Easy identification Potential stigma

"Malaria passports" or identification cards	A few	Personal record of testing and treatment history Costly to implement

Testing on arrival in villages	Some	Would detect unscheduled travel such as OBM Would require additional training of health workers or volunteers and community awareness

Legislation to enforce testing	Many	Empowerment for health workers and volunteers Reduce noncompliance

Most preferred immediate treatment for positive cases no matter where testing occurred, including on board the ships. Some suggested treatment initiated before or whilst travelling could make people seasick.

*' For pregnant mothers or young children it might not be wise for them to drink medication especially at sea otherwise they may get sea sick and throw up, and the medicine....won't be able to do its work in the body' *(Female participant, FGD village A)

An issue commonly raised by participants was that an education and awareness campaign about any intended surveillance-response system would be required and should be carried out intensively and with sufficient lead-time to implementation. Failure to do this may cause confusion and stress amongst travellers, and hence reduce both the acceptability of the intervention and passenger participation.

*'...much awareness must be given to these populations so that they will know, as long as people know they will cooperate.' *(Female participant, FGD village B)

In discussions about air travel, most participants reported the testing of passengers in Honiara before boarding to be acceptable and more feasible.

### RDT testing on unscheduled private transport

Many participants suggested that the Forestry Department and the logging companies should be responsible for RDT testing and surveillance of their own employees and some suggested that additional supporting legislation would assist to enforce their compliance. Many participants held negative views regarding logging operations in Isabel Province and considered logging camp workers a likely source of imported malaria cases.

*'That's the most people whom I'm suspecting of carrying malaria (the loggers), and spreading malaria' *(Male key informant, village E)

Most participants reported that people travelling by OBM into Isabel Province should be tested on arrival at their destination village. Almost all participants were of the opinion that identification of new arrivals in villages (known as "community eyes and ears" surveillance) would be straightforward because of the small size of all villages.

*'Villagers will be responsible to pick out a new face in the village and direct him or her to the place to get RDTs done. If the village is close to a health centre then go and get tested there. If no clinics nearby then someone in the village should be trained to do RDTs on these people' *(Male participant, FGD, village A)

### Community participation in malaria control

Most of the narratives from the participants describe strong bonds within and between villages in Isabel Province. The Tripod structure was reported by many participants to have a strong influence on communities with the spirit of togetherness in community tasks and activities frequently highlighted. The church, and organizations within it like the Mothers' Union, often assists provincial malaria team activities.

The study revealed a high level of knowledge about malaria transmission and prevention amongst participants and most participants mentioned the importance of continuing current prevention and control strategies [22]. However knowledge of the use of RDT for diagnosis and Coartem™ for treatment was not very high potentially due to their recent introduction as part of the new national treatment guidelines [23] and due to the very low malaria prevalence in Isabel Province.

The malaria prevention activity most frequently discussed and mentioned by almost all participants was the Tidy Village Campaign, where community members are encouraged to clean their village, including potential mosquito breeding sites, every Friday. This was originally initiated and run by health workers and the malaria team but now runs under the leadership of village chiefs and other community members.

*'Every Friday when the chief beats the drums or makes noise with the (loud) hailer and puts up the "malaria flag" the whole village will work with the village health members in cutting grass, clearing drains, and so on...' *(Male participant, FGD, village B).

## Discussion

Countries that have successfully achieved and maintained malaria elimination generally have an effective surveillance and appropriate response system in place to prevent disease re-introduction [14]. Many countries in which malaria has re-emerged after coming close to elimination have failed to maintain vigilant and robust surveillance [14,24]. As control programmes reduce the incidence of malaria, the pattern of disease also changes, necessitating extensive disease monitoring to determine the most efficient and cost-effective pre-elimination strategies [24]. The importance of maintaining surveillance is clear with examples of large malaria resurgence in Sri Lanka in 1968 [25] and in Madagascar between 1985 and 1990 after transmission had been interrupted [26] due to reduced surveillance and other control measures.

Adequate surveillance with appropriate response is essential for control and is of equal or more importance when countries or regions move towards elimination. One of the preconditions for the certification of malaria elimination by the WHO is the presence of a high quality surveillance-response system [27].

When countries or regions move towards elimination, monitoring and evaluation resources should trend away from the measurement of morbidity and mortality, which becomes increasingly difficult and insensitive, towards active case detection approaches as part of surveillance, followed by swift public health action to prevent foci of infections becoming epidemics [28,29]. The Malaria Elimination Group (MEG) goes further to define the key components that comprise an effective surveillance-response system [14,30].

It would be essential to engage the strong existing community structures in the design of a surveillance-response system in Isabel Province. Community participation in surveillance programmes in other countries has been shown to be of great benefit and contributes to the long-term sustainability of such a programme [31-33]. It has also been shown to be beneficial in other island nations and in particular in neighbouring Vanuatu where continuing community-based surveillance has helped to maintain elimination status on Aneityum island [31,33].

High levels of disease awareness in the community along with systems of timely case reporting allow the effective implementation of a participatory surveillance-response system which is more financially and logistically feasible than widespread screening systems requiring large amounts of manpower and resources [34,35]. This approach is important through the phases of elimination, from intensified control to 'holding the line' [35].

Whilst beneficial components for an effective surveillance-response system already exist in Isabel Province, the size of the task to implement an RDT based surveillance-response system must not be underestimated. With a population of around 26,000 people who travel extensively on a variety of modes of transport the preparation, planning, resources, time, human resources and funds required to set up the programme would be considerable. If implemented, it should be incorporated as an integral part of the SI National Malaria Control Programme to achieve sustainability and contribute to the strengthening of the national health system.

Since the study was performed, the Ministry of Health and the NVBDCP, SI have determined that screening all travellers entering Isabel Province at ports and airports is not currently viable due to prevailing financial and logistical constraints. Therefore it has been determined that a more informal system of surveillance by engaging community members to identify new arrivals, including returning Isabel residents, and direct them to malaria testing at the nearest health facility is more feasible [36]. It is noted that such a system of "community eyes and ears" has existed informally to some extent for years, but now a major awareness campaign throughout the province has been recommended by health authorities to more thoroughly educate residents about this system [36]. The responses given by our participants suggest acceptability and support for such a system. For this to be effective however RDT coverage will need to be scaled up and should be prioritized at health facilities.

When it becomes feasible for resources to be shifted towards a more formal surveillance process [28], a system based on RDT testing of new arrivals would be a necessary step towards more rigid surveillance. Solomon Islands can learn and be inspired by established malaria screening systems such as in international airport arrivals in Oman [37], Mauritius [14] and Zanzibar [38] and in some domestic airports in Vanuatu [33].

The implementation of such a system would require key logistical considerations such as attention to the high volume of passenger travel at holiday times and the busy and sometimes chaotic boarding and disembarking processes at the wharfs. Although less common, unscheduled inter-provincial travel by OBM poses numerous challenges for malaria surveillance. The risk that this type of travel poses on the population in Isabel Province is difficult to measure. The southern region of frequent inter-provincial OBM travel appears to overlap with areas previously identified as "hot spots" by the NVBDCP; however these areas were not corroborated by the most recent mass blood survey conducted in on Isabel Province in 2009 [8]. Suggestions towards surveillance raised by community members such as ideas regarding the timing of testing to minimize interruption to ticketing and boarding processes should be considered. Positive cases should be treated as soon as possible and followed up by the malaria team which was also suggested by many participants. It is advantageous to provide free treatment for all these cases [37].

Logging activity has been previously implicated in causing increased malaria prevalence due to the change in vector ecology and to population mobility [10]. Ideally logging camp managers should incorporate a system to ensure that all workers arriving on site from outside Isabel Province have had RDT done at some point in their journey. This implementation would require assistance from the provincial government for policy development and/or financial support.

The finding that travel to other provinces by OBM mostly occurs from two small coastal regions is an advantage for the implementation of a surveillance-response system however this type of travel still poses a significant challenge. An education and awareness campaign is an essential component to encourage compliance, to minimize confusion and to reduce stigma towards positive cases. Malaria elimination feasibility assessments in other countries have recommended that education and community involvement are essential in an elimination programme [38].

### Limitations

This study was conducted in five different villages in a relatively limited area of Isabel Province (due to logistical difficulties accessing villages further away) and was potentially not representative of the entire island. In addition, it is acknowledged that the capture of the full range of community views may have been reduced by selection of participants having been facilitated by village chiefs. However, as there was participation of key informants from across Isabel Province (as a result of the concurrent Isabel Provincial Assembly and Provincial Malaria team meetings in Buala), authors are confident that a sufficient range of perceptions and attitudes across the island were captured.

A key assumption in this study was that RDT-based surveillance programmes are an effective strategy to detect the imported reservoir of infection in this setting. The authors recognise that people with chronic low-grade infections, which may typically be characterised by gametocyte carriage, may not be detected by RDTs and yet represent an important reservoir for future malaria introduction and transmission in locations where transmission has been interrupted. In this situation, polymerase chain reaction (PCR) assays would theoretically be of value but clearly this would not be a viable option for active surveillance in rural settings in the South West Pacific. These issues require further scrutiny but were beyond the scope of this current research.

All FGDs were conducted in SI Pigin and the non-SI Pigin speaking facilitators conducted KIIs in English. Most key informants spoke English well but some may have had difficulty expressing views and opinions to their full extent due to their incomplete grasp of the language. This problem was overcome by recording sections of dialogue in Pigin and later translating it to English by the SI Pigin speaking facilitator.

Finally, data collected from Solomon Airlines and from the IDC provided some quantitative information regarding the number of passengers on flights and ships between Honiara and Isabel Province. The quality of data from the IDC was poor for some months, which resulted in limited understanding of the number of passengers travelling by ship. Unscheduled travel between provinces by OBM and by logging barges can occur almost anywhere on the island at any time and therefore quantitative data on this aspect is not recorded and impossible to obtain.

### Conclusion

In summary, a surveillance-response system to prevent introduction and re-introduction of malaria to Isabel Province would be feasible and acceptable to the community. The study found many factors that would contribute positively towards the feasibility of such a system. The challenges are less conceptual but are within the operational planning of the National Malaria Control Programme. This study is one of few that explore the feasibility and acceptability of a surveillance-response system along with human mobility patterns. It highlights how factors influencing migration often affect poor people in malaria endemic regions and that a greater understanding of population movement can improve malaria control activities [12].

Success in reducing malaria transmission to such low levels in Isabel Province has been attributed to broad community participation along with the early detection and effective treatment of malaria cases. These strengths form solid foundations for the proposed surveillance-response system, which in turn will be vital to achieving and maintaining malaria elimination in Isabel Province.

## List of abbreviations

AusAID: Australian Agency for International Development; FGD: Focus Group Discussion; IDC: Isabel Development Company; IRS: Indoor residual spraying; KII: Key Informant Interview; MEG: Malaria Elimination Group; NVBDCP: National Vector Borne Disease Control Programme; OBM: Out Board Motorboat; PacMISC: Pacific Malaria Initiative Support Centre; PCR: Polymerase Chain Reaction; RDTs: Rapid Diagnostic Tests; SI: Solomon Islands; SIDT: Solomon Islands Development Trust; SPH: School of Population Health; UQ: The University of Queensland; WHO: World Health Organization

## Competing interests

The authors declare that they have no competing interests.

## Authors' contributions

MOS, GK, YY, LL, AB, RW, JA and AV participated in the conception of study design. The field research activities were carried out by MOS, GK, YY and LL and were supported and supervised by RW, JA and MW. Data analysis was done by MOS, GK and YY with support and feedback by JA, MW and RW. Manuscript drafting was done by MOS with reviews and contributions by RW, JA, MW, AV and MT. All authors have read and approved the final manuscript.
